# What value for whom? – provider perspectives on health examinations for asylum seekers in Stockholm, Sweden

**DOI:** 10.1186/s12913-018-3422-1

**Published:** 2018-08-03

**Authors:** Sara Delilovic, Asli Kulane, Nina Åsbring, Anneli Marttila, Knut Lönnroth

**Affiliations:** 1 Department of Public Health Sciences (PHS), Social medicine, Infectious Diseases and Migration Research Group, Karolinska Institutet, 171 77 Stockholm, Sweden; 2 Stockholm County Council Health Service, Box 6909, 102 39 Stockholm, Sweden

**Keywords:** Asylum seekers, Health, Screening, Implementation, Provider perspectives, Health care access, Sweden

## Abstract

**Background:**

In Sweden asylum seekers are offered a voluntary health examination, free-of-charge (HE). The HE coverage is low. The organization and implementation of the HE involves collaboration between different agencies with different roles within the provision of health information and service. This study aimed to assess their perspectives on the barriers and facilitators regarding implementation of the HE, as well as on the purpose, content and value of the HE.

**Method:**

Thematic analysis of focus groups, individual and group interviews conducted between 2016 and 17 with 41 participants from various authorities and healthcare professionals involved in the delivery of HE in Stockholm.

**Results:**

Suggestions were taken from interviewees of how to facilitate the uptake and delivery of HE through improved outreach to the target group with better collaboration, coordination and continuity between authorities. Apart from control of specific communicable diseases, the perceived ultimate goal of HE varied and was often vaguely formulated. Respondents desired better monitoring to assess the effects of HE and predict needs among asylum seekers. This included standardized procedures to promote equitable health care access and more explicit inclusion of mental health and other health dimensions in the HE.

**Conclusion:**

There are several possible avenues for improving HE coverage and uptake. However, ambiguity exists concerning the benefits of such efforts given the uncertainty of the value of HE. Lack of available data on health status, determinants of health and impact of HE among asylum seekers emerged as barriers preventing optimal approaches for the assessment of health needs. Implementation of standardized guidelines, procedures and documentation would aid the understanding. A more holistic approach beyond infectious diseases is necessary. This would only be useful if there is value in screening for such conditions. More research is required to assess the effectiveness and cost-effectiveness of HE and related screening policies in Sweden.

**Electronic supplementary material:**

The online version of this article (10.1186/s12913-018-3422-1) contains supplementary material, which is available to authorized users.

## Background

### Migration and access to health care

In recent years, war, conflict and violation of human rights have led to massive forced displacement, with millions of people seeking protection in other countries [1]. During 2015–2016, Europe experienced an influx of people seeking asylum from multiple nations with Syria, Afghanistan and Iraq being the top three. [2]. Sweden and the rest of Europe have a humanitarian responsibility for these people, to assure their safety and uphold their human rights to health and access to healthcare services [1, 3]. According to the UN definition, a refugee is “*a person who is outside his or her home country, has a well-founded fear of persecution due to his or her race, religion, nationality, membership of a particular social group or political opinion, and is unable or unwilling to gain protection from that country, or to return there for fear of persecution*” [4]. An asylum seeker is a person who files for a refugee status in the host country and awaits a decision. Compared to refugees, asylum seekers in Sweden have not been granted a residence permit and therefore they are not included in surveillance or health information systems [1].

International law protects the human rights of refugees and asylum seekers. The international covenant on social, economic and cultural rights is a key legal document ratified by 164 countries, including Sweden [5]. The 1951 refugee convention states that refugees should “enjoy access to health service equivalent to that of the host country” [4]. The Governments faces challenges in taking human rights, including right to health care into national plans, policies and strategies. Inadequate entitlement and legal restrictions to use health care services amplifies health inequalities leading to the violation of human rights of the migrants [1, 3, 6, 7]. The absence of restrictions does not imply equity. Differences in access to health care for migrants are shaped by language barriers, sociocultural factors, and the migrants’ lack of awareness of available health services, cultural barriers and structural barriers [6, 8]. To respond adequately to health and social care needs of refugees and asylum seekers and improve accessibility and quality of care, collaboration and intersectoral communication between authorities and actors such as, health service providers, non-governmental organizations and all levels of government (national, regional and municipal) is essential [9, 10]. Furthermore, specific health needs of refugees and especially asylum seekers are poorly understood as high-quality data on determinants of health, health status and utilization of health services are rarely collected [1, 6, 9, 10]. To respond to the need of refugees and asylum seekers, adequate monitoring and research can assist in acquiring relevant health information that could lay the foundation for interventions and public health initiatives aiming to improve service utilization, access and health outcomes [1].

### Screening and health examinations

Migration has raised concerns about the transmission of infectious diseases. Screening programmes targeting newly arrived asylum seekers are common in host countries as a means to minimize transmission [6, 8, 11]. Screening newly arrived refugees has been criticized as ineffective, discriminative and of contestable value. There is no clear evidence of individual and public health benefits or cost-effectiveness of such procedure and it remains open to considerable doubt [11–13]. Despite this, medical screening exists in many countries. There are differences in practice and implementation in terms of timing of the screening, location, specific medical screening programme (mental and physical) and whether it is done on a voluntary or compulsory basis [8, 14]. Screening practices in the European Union (EU) region more often focus on communicable diseases while mental health screening is only offered in 11 EU countries (three compulsory, eight voluntary) [8, 14]. Focusing on infectious diseases might not accurately reflect health needs. In addition, legal access to health services vary between countries. Countries such as Denmark, Norway and The Netherlands grant asylum seekers unconditional entitlements to the same range of services as nationals/citizens while in other countries such as Sweden, it is more restrictive [8, 12–14].

### Health examination in Sweden

During the period 2015–2016 almost 200,000 people sought asylum in Sweden [15]. Asylum seekers in Sweden are entitled to necessary medical care as provided by the Swedish reception of asylum seekers’ Act (LMA). This includes emergency or urgent medical and dental care, maternity and childbirth care, contraceptive advice and abortion. Children asylum seekers under the age of 18 are entitled to the same health care as natives. Asylum seekers who are granted a residence permit, referred to as newly arrived refugees are entitled to the same healthcare as other citizens [16]. By law, all asylum seekers must be offered a free-of-charge health examination (HE) by the region/county where they reside. Newly arrived refugees are entitled to a HE within 1 year of receiving a residence permit. Other migrants such quota refugees and refugees coming on family reunification are entitled to a HE, however in this case, the examinations are neither systematically offered, nor regulated in the law in the same way as for asylum seekers [16, 17]. Data shows poor participation within the HE, with less than 50% of asylum seekers undergoing the HE [18]. Limited research has been carried out investigating reasons behind this, however structural and organizational shortcomings have been suggested [19, 20]. Studies have shown that poor communication, inadequate information and lack of clarity regarding the purpose of the HE, ambiguity and mistrust could to some extent explain the poor attendance [19, 20]. Asylum seekers in Sweden felt they received insufficient information both ahead and during the HE, perceived health needs were not met and focus was partly given to infectious disease control [20]. Other studies have found that low levels of comprehensive health literacy, individual’s competence in accessing, understanding, appraising and applying health information was negatively associated with the quality of the communication and usefulness of the HE [21].

In Sweden, responsibility for health care is shared by central government, county councils and municipalities. The central government, through the Ministry of Health and Social affairs is responsible for the overall health policies. County councils, together with the municipalities, operate autonomously and they hold the responsibility for public services across various sectors. The principle of local self-government gives the county councils and regions the right to design and structure their activities in the light of local conditions [22].

The organization around the HE involves different entities. The content and recommendations are regulated by the National Board of Welfare and the Public Health Agency and represent the minimum level. Each county council can develop their own templates for the HE and the content can vary widely across and within counties and between health care providers [23]. The Migration Board is obliged to inform the asylum seekers about the right to a HE and health care providers are responsible for the invitation and provision [24]. Training is provided to health care professionals conducting the HE, however this varies and literature shows there is a need for more education with regards to transcultural nursing and cultural competence [23]. The purpose of the HE is to: identify mental and physical health needs demanding care, in line with concept of “care that cannot be postponed” and to detect and control for infectious diseases such as human immunodeficiency virus (HIV), hepatitis and tuberculosis [17]. The examination should, ideally, include a discussion about health issues in addition to testing for infectious disease which is regulated by the National Prevention and Control strategy. Guidelines and indications for screening depend on infectious disease epidemiology in the country of origin of the asylum seeker/newly arrived refugee [25].

In Stockholm County there are 7 designated health care centers responsible for the provision of HE for asylum seekers and newly arrived refugees.

In this study, we aimed to investigate perceptions and attitudes among actors involved in the implementation process of the HE and health care professionals carrying out the HE in Stockholm County concerning barriers and facilitators of implementing the HE, as well as on the purpose, content and value of HE.

## Methods

### Design

We used a qualitative design since this is considered valuable for exploring areas not extensively studied and was judged to be the most suitable approach with respect to the research question [26, 27]. Interview guides were developed, one for the health care professionals and one for the actors, with focus on specific areas such as: information and access barriers for asylum seekers; information and coordination gaps between authorities; content, quality and value of the HE; and monitoring and evaluation of the HE (Additional file 1). Domains for the interview guides were generated from previous research, based on existing literature and the authors’ personal and professional experiences [12, 18, 20, 23]. To finalize the guides, they were tested on colleagues experienced in providing migrant health care.

### Study setting and sampling

A non –probability, purposive sampling was used where interviewees were recruited on the basis of their occupation, previous work and involvement in the implementation process of the HE. They were either directly or indirectly involved in the implementation and had different functions such as policymakers, government officials and/or healthcare professionals. The chosen government officials and policy makers held positions relevant to migration and health, they were considered as the most appropriate individuals to address the research questions.

At the time of the study, health care professionals were practitioners working at one of the seven health care centers conducting HEs. All interviews were carried out in Stockholm County and participants were initially contacted by the authors via phone or email and invited to participate in the study.

### Participants

The interview sample comprised 41 participants (23 health care professionals, 1 administrator at one of the health care centres and 17 policy and government officials) from the following agencies/authorities; The National Board of Health and Welfare, The Public Health Agency of Sweden, County Administrative Board, Swedish Migration Agency, Sweden’s Public Employment Agency, Swedish Association of Local Authorities and Regions (SKL), Transcultural Center and health care professionals from Stockholm county.

### Data collection

Data was collected from September 2016 to January 2017 using two focus group interviews, three group interviews and 20 individual semi-structured interviews. The teams of health care professionals working in each health care center included up to seven people. To capture the experience in each center, focus group interviews were considered a suitable approach. In five centers, between one and three individuals oversaw the HE and therefor individual interviews and group interviews were conducted. Each focus group included seven participants, two group interviews included two to three participants. In the group interviews, each participant took turns in answering the questions. For policy and government officials, individual, in-depth interviews were conducted. Informants from the policy/government category were viewed as proxies for their respective organization or institution or they were the only ones working with migration and health at institutional level. To foster reflexivity, multiple researchers were included in data collection [26]. The interviews were conducted in Swedish by SD, AM, and NÅ and took on average 60 min. A respondent- validity seminar was held in March 2017, where all participants were invited to discuss the findings. This technique involved testing the preliminary results with the participants to be able to assure reliable interpretation, check authenticity and to refine the understanding [28].

### Data analysis

A thematic analysis was conducted [29]. All interviews were digitally recorded and transcribed verbatim. Analysis was guided by the different phases of thematic analysis involving the following steps: familiarization with the data (reading and re-reading the data, noting down initial ideas); generating initial codes (systematically identifying items and repeated patterns); identifying themes (categorizing codes into potential themes across the data set); reviewing themes (generating a thematic map, assuring coherent patterns and testing the themes in relation the data set) and labeling themes (refining specifics of each theme and generating names) [29, 30]. Analysis was performed by the researcher (SD) in collaboration with co-authors (AK, NÅ, AM), analysis was discussed, refined and issues and reflections were deliberated, to confer rigor [26, 31]. The data was analyzed as a whole and not stratified by professional category (government and policy officials/health care professional).

### Ethical considerations

Ethical clearance was obtained from the regional ethics board of Stockholm County (2016/1648–32). Written consent was obtained from all participants after introducing them to the study. Participants were provided with information regarding their anonymity and secure data processing. Respondents were also informed about their right to withdraw their participation at any time without further explanation. In the presentation of the findings, all quotes are anonymized.

## Results

Our findings focused on perceptions and attitudes among entities involved in the implementation of the HE and health care professionals carrying out the HE. Three main themes were identified presenting issues associated with the HE, communication and information provision challenges and challenges in coordination. Themes are presented with categories reflecting different aspects of each theme, giving a more detailed explanation of the results. Each theme and its subsequent categories will be presented discussed below.

### Health examination in practice

The theme explains respondent’s views on the practicality of conducting the HE and its coverage in terms of recipients, objective, content, scope and its function. This theme illuminates complex challenges with responding to migrant’s needs, delivery of health services and access to health care.

### Ultimate purpose of HE

The health examination was considered by the respondents to be beneficial and of value for both the individual and the society. However, there were divergent opinions with regards to the purpose of the HE. The Swedish Board of health and Welfare states that the purpose of the HE is to identify physical and mental health needs requiring immediate care, and to assess the need for health care services and preventive measures for infectious diseases. Some respondents expressed that the way the HE is practiced today, focused heavily on infectious disease identification and control rather than giving attention to other health conditions.

*“I mean, what we are doing is more in the area of screening so it’s not that in depth at all, considering the mental part …we can help them later, refer to a physician […] Unfortunately it’s like that, I think there should be more space for that and maybe more specific also, what should be asked” (group interview 3).*

*“It is probably more control for infectious diseases, it’s my opinion whether it is right or not, I think it still remains there” (interview 3).*

The respondents further expressed the challenges with the concept of “care that cannot be postponed” as it entitles asylum seekers to limited health care services and how this could be questioned considering the right to health care and equality. For health care providers these aspects created a dilemma when there is no guarantee for follow up and it was said that different providers assess “care that cannot be postponed” differently. This was especially problematic with families, where the children have the same rights as citizens but the parents are not entitled to such extensive health care services.

*“Everybody looks at it differently. It is really different from doctor to doctor. And it also depends on where you go and who the doctor you met is... One of our physicians she doesn’t see a difference between those who have a social security number and don’t, but then there are those that think a bit differently” (group interview 2).*

### Potential groups not covered

Another highlighted challenge was that the HE was not offered to all migrants. Most migrants who come on the premise of family reunification do not go through the same process as asylum seekers and are not offered a HE. Even though many come from similar circumstances, they are not invited to a HE.

*“Refugees coming on family reunification don’t go through the same process as asylum seekers […] I have seen it many times, many who come with their children and they don’t know anything because no one informed them” (interview 10).*

### Going beyond infectious diseases

The majority of the respondents addressed the question of mental health, expressing there was a need to change focus to mental health by assuring that questions of this character are included in the HE. Some proposed that in addition to mental health issues, standard procedures to identify sexual and reproductive health, hearing and sight issues should be a part of the HE. Health care providers explained that they sometimes use simple indicators such as sleeping habits to try and capture aspects of mental health. They often described the challenge between trying to assess mental health issues indirectly, without asking direct questions, when at the same time respecting the migrant’s refusal to not answer. Time is needed to discuss traumatic events and several pointed out the importance of having a transcultural perspective when dealing with mental health of migrants.

*“In the template we use, mental health is missing a bit, but sometimes it emerges during the conversation…how you sleep, how you function… eating habits. If you notice something maybe you continue ask about it, if it’s okay…but if they don’t respond you don’t go in-depth […] You notice if they want to talk about it or not” (focus group 1).*

*“It’s not specified, there is one heading [in the templet] titled mental health but then it’s up to you how you formulate the question” (group interview 1).*

### Effects not known

Several respondents expressed that there was a need for further developing the content, design and monitoring. One of the main challenges was to assess health care needs among the group as data was not collected in a systematic way. It was said to be difficult to estimate and evaluate the impact of the HE for public health as neither results of the HE, nor health status after HE were monitored.

*“We know very little about the effects of the health examination, but we can at least follow up and control infectious diseases” (interview 3).*

### Quality assurance

Respondents emphasized it was necessary to consider some structural changes around the templates used for the HE. Health care professionals use different templates which lead to differences in practice. Some said that implementing standardized templates to harmonize the content and procedure would be valuable in many aspects. This could also increase data availability on determinants of health for asylum seekers, reduce variation practices among health professionals and enable quality assurance and evaluation.

*“One suggestion is that all health care centers that conducted health examination with asylum seekers should use the same template […] I mean when we document [in the electronic medical system], we document with different words and if you want to use that data for statistical purposes example, how are women’s wellbeing,…you can’t get that type of information because we do it so differently” (focus group 2).*

### Challenges in information and communication

The theme explains various obstacles and challenges in information and communication flow regarding the HE to asylum seekers and newly arrived refugees. It also illustrates methodical challenges with reaching out to asylum seekers and newly arrived refugees while assuring cultural competence.

### Logistical, linguistic and cultural barriers

Willingness to participate in the HE among asylum seekers and newly arrived refugees were reported as difficult to assess, however, participants listed logistical, linguistic and cultural barriers that might be contributing to the low participation rate.

*“Asylum seekers who live in accommodations provided by the Migration board have access to everything, they have personal in place and other staff from the migration board coming to them. But all the others, those who live in their own accommodation they end up so far from all information” (interview 5).*

The initial information about the right to undergo a HE is to be given by the Swedish Migration board, however there seemed to be concerns among some respondents about the content of the information and some said asylum seekers sometimes think the HE is a part of the asylum process, and its mandatory in order to obtain a residence permit.

*“And some thinks it’s mandatory to do the health examination. I have heard several say…I have to do this because the migration board said so” (focus group 2).*

Apart from the language barrier, there were some who felt that there were cultural barriers, and that there might be mistrust among migrant groups about how Swedish authorities operate, and this mistrust and doubt could affect how they perceive and interpret the information.

*“There are many who are ambiguous and skeptical about the migration board and the state in general, which is understandable because of different circumstances people come from” (interview 12).*

### Need for competence development

Some of the non-health care respondents who were responsible for the provision of initial information to asylum seekers and newly arrived refugees perceived they had a lack of knowledge about what the HE comprises. They expressed it would be valuable if they had more specific information about the HE to be better convey this to the asylum seeker. Health professionals said this corresponded to their experiences when sometimes individuals showed dissatisfaction with the content of the examination as it did not satisfy their prior expectations.

*“They think they are going to get everything checked, physical examination and all possible laboratory tests…then we will have to say…no unfortunately, this it much more limited” (group interview 1).*

*“I don’t feel that I can give detailed information, I can’t say what’s being offered. Probably I wouldn’t talk about it that much, I would tell them it’s a general checkup” (interview 5).*

The lack of knowledge among migration authority staff regarding the actual content of HE was mirrored by a lack of knowledge among health staff about the asylum application process. Health care professionals described a need both for general information about the asylum process, which would have been helpful in the first meeting with the asylum seeker, as well as better culture competence. Some lacked training and knowledge on how to deal and communicate health related issues, especially mental health with people from different cultures and backgrounds.

*“I don’t think we get so much information about the actual asylum process and what they go though. [...] Sometimes they ask some specific questions and we can’t answer because we don’t know” (focus group 1).*

### Lack of coordination

The theme highlights collaboration and coordination issues around the HE from an organizational perspective. It explains challenges with patient safety and interaction between different professionals.

### Many actors - poor collaboration

Respondents emphasized the importance of collaboration, though they felt there was a lack of implemented directives to achieve this. This was mainly hampered by laws, regulations and how each authority had to relate to their tasks/responsibilities.

*“Coordination is really important and therefore we need to cooperate and coordinate between actors, between county and municipality. It’s something that requires resources, personally I feel it’s needed and should be addressed. [...] There is more to do, to share more information and become more efficient” (interview 3).*

The level of collaboration and co-ordination between actors varied. Several respondents experienced barriers and these were mainly derived from a lack of communication, awareness and recognition of the role of each profession. Others perceived collaboration with the county council, municipalities and other stakeholders as challenging. In Sweden municipalities are responsible for social care while county councils are responsible for health care.

*“It’s a huge problem that the rest of the health care don’t know what a HE is, other [health care professional] refer their patient to us and some patient even say they have been told they have to visit us before they are eligible for other health care. It is noticeable that there is a lack of knowledge among others in the health sector […] I think all primary health care centers within Stockholm county should get some information” (focus group 1).*

### Coordination identity number

Respondents highlighted the need of a coordination number for asylum seekers to ensure better monitoring of previous health care services. Currently, each county has a separate system for temporary identification numbers, one individual can receive several numbers, and these are not linkable across counties and municipality services. A coordination number is expected to increase patient/client safety, facilitate contact between health care professionals and contribute to better provision of relevant and necessary information.

*“I think the patient safety would increase and it will also be easier to find the patient and make sure it’s the right patient” (interview 20).*

*“One might have been treated for mental health problems during the asylum period and that is nothing that we are informed about, it would be much better if we knew that from day one because then we would be able to help people easier […] It would facilitate the process for us if we knew which actors have been in contact with the individual” (Interview 14).*

## Discussion

This study highlights challenges in the structure, coordination and access to health care for asylum seekers and newly arrived refugees, as well as other factors hampering the implementation of the HE. The results show there is a lack of clarity regarding the objective of the HE. The official purpose of the HE is to identify health needs both of mental and physical character, to detect and control infectious diseases, and to provide general information about the health care system [17]. The patient interview which is one of the main parts of the HE “should bring out and examine past and current mental health, psychosocial situation and the experience of traumatic event (victim or witnessed violence, torture or sexual assault)” [17]. However, a common perception among respondents was that in practice insufficient attention is given to mental health problems and other health needs beyond infectious diseases. Studies have shown that migrants themselves experience the HE as a control of infectious diseases. It has been argued that it is unacceptable that other health needs are overlooked and inadequately addressed [12, 13, 19, 20, 32]. The problems health care professionals encountered highlight some potential sources for the lack of mental health assessment, such as: intercultural competence, guidelines and tools on how to appropriately assess mental health and individuals’ willingness to disclose personal experiences. However, expanding the HE and improving coverage is only meaningful if we know that screening for such conditions is effective and cost-effective, which we do not [6, 8, 11–13]. Additionally, it would only be useful to prioritize if care needs are appropriately met and help is provided for identified problems, which may be a challenge as asylum seekers are only entitled to “care that cannot be postponed” and emergency care, turning their migration status into a factor for entitlements to health care. Sweden provides limited access to health care for asylum seekers, revealing major discrepancies with human rights law [4, 5, 33], while current policies are somewhat ambiguous and raise ethical dilemmas for health professionals providing care for asylum seekers. In addition, not offering the HE to all migrants, irrespective of their status (asylum seeker, quota, family ties) might be misinterpreted as not being in need of a HE and might inhibit access to health services.

Furthermore, data and information on health status, health determinants and the extent to which asylum seekers get access to needed services, in line with “right to health” principles is limited [23]. Data is not collected on a routine basis and the absence of standardized interview guides on how to carry out the HE leads to variation in practice among health professionals. While Sweden has a long tradition of building excellent health databases, there is a long way to go to improve systematic data collection and monitoring of HE and health care for asylum seekers in general. Amassing data in a systematic way and implementing a standardized guide would not only lead to more standardized health examinations, it will also form the basis of operational research which would help to assess future needs, improve implementation scale up and enhance quality [1].

Providing comprehensive health-related information to asylum seekers and newly arrived refugees is of vital importance. Previous studies have shown that lack of motivation among asylum seekers to participate in a HE does not seem to be the main cause of low coverage. Rather than rejecting the invitation, low participation was explained by structural barriers such as not receiving the invitation or lack of comprehensive information to make informed choice about their health [19, 20, 32, 34]. Increasing the awareness of available services and assuring geographical accessibility using a multi-sectoral outreach approach can assist in providing health-related information [6]. In addition, ensuring education for personnel meeting migrants and making sure information is delivered in a culturally and linguistically appropriate way could mitigate cultural and structural barriers [13, 35].

In all public health initiatives it’s important to adopt a multilevel approach and recognize the fact that health systems are made of many levels and by many actors, which by definition involve more than the sum of a single individual organization [14]. The lack of cooperation within and between health care and other actors was emphasized by the respondents as a challenge for effective and efficient outcomes. The National Board of Health and Welfare recently assessed that collaboration and coordination between health care and other actors involved in health care for asylum seekers is lacking. Coordination needs to be improved to better respond and meet future needs of asylum seekers and newly arrived refugees [23].

### Limitations

The interviewees were, apart from the health care professionals, recruited from a broad range of different authorities and organisations, working on local, regional and national levels. This triangulation method enabled us to better visualize the phenomenon studied [36]. However, there is a possibility that other individuals, whose perspective is of value, have been missed in the selection process. Other institutions and non-governmental organizations (NGOs) in contact with asylum seekers and newly arrived refugees could have provided valuable input. Nevertheless, health care professionals were recruited from all seven health care centres conducting HE, allowing information to be checked across informants, which make it a comprehensive study for Stockholm County. However, the situation is likely different in other regions. Interviews were transcribed verbatim by the same researchers who conducted them and quotes were used, which strengthens the validity and shows the logic behind the interpretation of the data [36]. Furthermore, interviews were conducted by different researchers which diminish the risk of personal biases such as attitudes, prejudices and personal experiences [26]. Respondent-validity was assured, participants were invited to a seminar where they had the opportunity to correct errors, challenge incorrect interpretations and critically analyze the results. [28]. Generalizability often referred to the extent to which the findings can be transferred to other settings or groups [31]. In the present study, we have studied health examinations performed within Stockholm County, findings may not be applicable in other counties and regions in Sweden as well as in other countries with different screening programs.

## Conclusion

Respondents reported several practical problems but also possible solutions for how to improve HE coverage. However, this study has identified more fundamental questions about the value of HE and how they could help guarantee “right to health”. On one hand, there are strong moral reasons for providing access to health care for all groups of migrants irrespective of their legal status. It also seems to make sense from a public health perspective. It is therefore plausible possible that legal entitlements should be expanded. On the other hand, there is insufficient evaluation data to judge if HEs are either effective and/or cost-effective health interventions. Their impact on infectious diseases, access to health care and equity require further study. It is recognized that asylum seekers and newly arrived refugees form a particular vulnerable group who require access to health care at a very crucial time in their lives. Right to access health care is fundamental, but the question remains if HE is a valuable element of such rights.

## Additional file

Additional file 1: Interview guides for health care professionals and authorities. (DOCX 18 kb)

## Abbreviation

EU: European Union
HE: Health examination
HIV: Human immunodeficiency virus
LMA: Swedish reception of asylum seekers’ Act
NGO: Non-governmental organizations
SKL: Swedish Association of Local Authorities and Regions
