# Supplementary material for: What value for whom? – provider perspectives on health examinations for asylum seekers in Stockholm, Sweden
Source: BMC Health Serv Res. 2018 Aug 3;18:601. doi: 10.1186/s12913-018-3422-1 (PMC6091028; doi:10.1186/s12913-018-3422-1)
Supplement: Supplementary file 1 — Interview guides for health care professionals and authorities. (DOCX 18 kb) [file 12913_2018_3422_MOESM1_ESM.docx]

**Guide Healthcare professionals**

**Presentation and background**

Please describe yourselves, your roles and responsibilities as practitioners at this health care center?

(Ex: how many HE do you perfume per month/your professional background/ how long you have worked here and so on?)

**Intro**

How are the health examinations performed here at this health care center? (Families/individual/mobile clinic)

Could you elaborate about the administrative work/practicality of inviting migrants to the HE?

(How do you know who to invite/how do you invite them (text message/letter/other channels?)

Do you send reminders?

Do you have suggestion for improvement, regarding the invitation process?

Which migrants groups are offered and invited to a HE?

What are your experiences of conducting the HE? Suggestions for improvement?

**Content of the HE**

In terms of the content, what are you experiences of the HE?

Do you use specific templates? Please elaborate

Is everyone using the same templet? Please elaborate

Is the templates useful? Please Elaborate

What are your thoughts about it - How well does HE cover the individual's need for health and health care, specifically for:

- Somatic illness/conditions/diseases
- Mental health
- Unhealthy lifestyle behaviors
- Infectious diseased

Do follow ups occur?

What kind of treatment is offered if there is a need?

**Care that cannot be postponed**

How is the collaboration with other health care instances/referral units?

Is it clear what “care that cannot wait” implies?

What are you experiences of defining and using “care that cannot wait” in practice? Please elaborate

How do you interpret the definition?

How is this documented?

**Goal and expectations**

What do you perceive as the aim of the HE?

How do you perceive migrants understanding of why they undergo a HE?

Do you feel that the HE meets their expectations? If not, why? Do you have any suggestions for improvement?

**Competence**

Do you experience that you have received/you have adequate education within the field of migration and health (issues related to migration)?

Do you get guidance in working with migrants groups and migrant health?

Based on your experience, what are the areas you would like to develop more skills in?

**Value**

How do you perceive the benefits with the HE? Value for the individual? Societal value/Public health value?

**Final Questions**

Do you have any general suggestion on how the HE could be improved?

What are the best and worst thing working with this area?

Is there anything else you would like to tell/share, that I haven’t asked you?

**Guide Authorities**

**Presentation and background**

Could you tell me about yourself, your background and how your work is related to asylum seekers, newly arrived refuges and migration health.

**Value, information, challenges, opportunities**

How is the offer for a HE communicated to the asylum seeker/newly arrived refugees

Can you tell us about the content of information?

Is information given about HU to all groups irrespective of migration status (asylum/quota/and so on)?

How do you perceive that the asylum seeker/ newly arrived refugees perceives the information?

What do you experience affect asylum seekers /newly arrived refugees willingness to participate or not participate in a HU?

What benefit/value do you think the HE has for migrants, irrespective of status?

What benefit/value do you think the HE has for the society?

Do you work to secure participation in HE? If so, elaborate how?

How do you feel that cooperation and communication with other actors involved in the reception of asylum seekers/newly arrived refugees work with regard to HE? Elaborate

- Is there efforts for collaboration?

- What information is provided between actors involved in different stages of the HE?

Do you have suggestions for improvement measures regarding the transfer of information about HU?

What strategies and improvement measures do you regard as valuable for increasing participation and interest in HU among asylum seekers/newly arrived refugees?

What would you consider to be valuable in the development work with HU?

In terms of human right and right to health care, what is your perception about the concept of “care that cannot wait”? Elaborate

**Final Question**

Is there anything else you would like to tell/share, that I haven’t asked you?
